# Ciprofloxacin enhances the biofilm formation of *Staphylococcus aureus* via an *agrC*-dependent mechanism

**DOI:** 10.3389/fmicb.2023.1328947

**Published:** 2023-12-21

**Authors:** Zhao-xia Luo, Yuting Li, Mei-fang Liu, Rui Zhao

**Affiliations:** ^1^Department of Clinical Laboratory, Medical Center of Burn Plastic and Wound Repair, The First Affiliated Hospital of Nanchang University, Nanchang, China; ^2^School of Public Health, Nanchang University, Nanchang, China

**Keywords:** Staphylococcus aureus, ciprofloxacin, Agr, agrC, biofilm

## Abstract

*Staphylococcus aureus* readily forms biofilms on host tissues and medical devices, enabling its persistence in chronic infections and resistance to antibiotic therapy. The accessory gene regulator (Agr) quorum sensing system plays a key role in regulating *S. aureus* biofilm formation. This study reveals the widely used fluoroquinolone antibiotic, ciprofloxacin, strongly stimulates biofilm formation in methicillin-resistant *S. aureus*, methicillin-sensitive *S. aureus*, and clinical isolates with diverse genetic backgrounds. Crystal violet staining indicated that ciprofloxacin induced a remarkable 12.46- to 15.19-fold increase in biofilm biomass. Confocal laser scanning microscopy revealed that ciprofloxacin induced denser biofilms. Phenotypic assays suggest that ciprofloxacin may enhance polysaccharide intercellular adhesin production, inhibit autolysis, and reduce proteolysis during the biofilm development, thus promoting initial adhesion and enhancing biofilm stability. Mechanistically, ciprofloxacin significantly alters the expression of various biofilm-related genes (*icaA*, *icaD*, *fnbA*, *fnbB*, *eap*, *emp*) and regulators (*agrA*, *saeR*). Gene knockout experiments revealed that deletion of *agrC*, rather than *saeRS*, abolishes the ciprofloxacin-induced enhancement of biofilm formation, underscoring the key role of *agrC*. Thermal shift assays showed ciprofloxacin binds purified AgrC protein, thereby inhibiting the Agr system. Molecular docking results further support the potential interaction between ciprofloxacin and AgrC. In summary, subinhibitory concentrations of ciprofloxacin stimulate *S. aureus* biofilm formation via an *agrC*-dependent pathway. This inductive effect may facilitate local infection establishment and bacterial persistence, ultimately leading to therapeutic failure.

## 1 Introduction

*Staphylococcus aureus* is a formidable pathogen responsible for significant hospital-acquired infections such as bloodstream infections, pneumonia, and surgical site colonization (Donlan, 2002). Its remarkable ability to form robust biofilms on host tissues and medical devices enables evasion of immune clearance and establishment of persistent, chronic infections (Conlon, 2014). Biofilm-associated *S. aureus* infections are notoriously difficult to treat, frequently involving implanted devices, and often leading to severe complications like bloodstream dissemination (Conlon, 2014). However, few drugs effectively eradicate mature biofilms, requiring concentrations 1,000-times higher than for planktonic cells (Lauderdale et al., 2010). This challenge is exacerbated by increasing multidrug resistance in *S. aureus* against methicillin, fluoroquinolones, and macrolides (Li et al., 2023; Tran et al., 2023). Understanding *S. aureus* biofilm formation is critically important given its role in antibiotic resistance and treatment failure.

Ciprofloxacin, a member of the fluoroquinolone family, traditionally is used to treat Gram-positive and Gram-negative bacterial infections by disrupting DNA gyrase and topoisomerase IV (Serizawa et al., 2010; Sharma et al., 2017). However, the emergence of increasing ciprofloxacin resistance among clinical isolates has become a major concern (Dalhoff, 2012). During antibiotic therapy, resistant bacteria and biofilm-embedded cells inevitably encounter subinhibitory concentrations of antibiotics (Kaplan, 2011). Subinhibitory antibiotics can function as signal profoundly influencing biofilm architecture, virulence factors, and gene expression patterns, though underlying mechanisms remain unclear (Atshan et al., 2021; Chen et al., 2021). Different antibiotics may have varying effects mediated through distinct pathways (Atshan et al., 2021; Chen et al., 2021). Subinhibitory concentrations of β-lactams notably upregulate *S. aureus* exotoxins and adhesins (Chen et al., 2021).

The effect of ciprofloxacin on *S. aureus* biofilm formation remains unknown. This study aims to investigate the impact of subinhibitory concentrations of ciprofloxacin on *S. aureus* biofilm formation and simultaneously elucidate potential mechanisms. This research offers valuable insights into optimizing antibiotic usage to enhance the efficacy of treatment for device-associated and recurrent *S. aureus* infections.

## 2 Materials and methods

### 2.1 Isolate identification and culture

Five clinical *S. aureus* isolates (SA001-SA005) were collected from the First Affiliated Hospital of Nanchang University and are listed in Table 1. Among the isolates, two were MSSA, and three were MRSA, derived from sputum, blood, and pus samples. MLST analysis identified SA001 and SA002 as ST5, SA003 as ST239, SA004 as ST59, and SA005 as ST7. *S. aureus* was cultured in Tryptic Soy Broth (TSB) with agitation at 220 rpm at 37°C unless otherwise specified. *Escherichia coli* (*E. coli*) was cultured in Luria Bertani Broth (LB). The following antibiotics were added to the medium when required: ampicillin at 100 μg/mL; chloramphenicol at 10 μg/mL. Ciprofloxacin (purity: ≥ 98%) was purchased from Aladdin (Aladdin, Shanghai, China) and dissolved in sterile water. Ciprofloxacin was incorporated into the culture media to explore the impact of subinhibitory concentrations of ciprofloxacin on the phenotypes of *S. aureus*.

**Table 1:** Characteristics of clinical *S. aureus* isolates used in this study.

Bacterial strains	MSSA/MRSA	Source	MIC of CIP (μg/mL)	MLST
SA001	MSSA	Sputum	0.5	ST5
SA002	MSSA	Blood	0.25	ST5
SA003	MRSA	Sputum	0.5	ST239
SA004	MRSA	Sputum	0.5	ST59
SA005	MRSA	Pus	0.5	ST7

### 2.2 Assessment of minimal inhibitory concentration

The MIC was determined using the microdilution method following the Clinical and Laboratory Standards Institute (CLSI) guidelines (Pierce et al., 2023). Briefly, ciprofloxacin was serially diluted 2-fold in 96-well plates containing Mueller-Hinton (MH) broth. Overnight *S. aureus* cultures were diluted in MH broth to 1.5 × 10^6^ colony-forming units (CFU) in a 1 mL volume, which was subsequently added to the plates. The ciprofloxacin concentrations ranged from 0.0625 to 128 μg/mL. No drug group was added as the control (no ciprofloxacin) and blank MH broth served as a negative control. After incubation at 37°C for 24 h, the experiment was repeated three times to ensure accuracy. MIC results were interpreted according to the CLSI (2023) guidelines for *S. aureus*. Quality control was performed by testing the ATCC29213.

### 2.3 Biofilm formation assay

The effect of ciprofloxacin on *S. aureus* biofilm formation was analyzed through crystal violet staining using 96-well polystyrene microtiter plates, as described previously (Xiao et al., 2023). Overnight *S. aureus* cultures were diluted in TSBG (Tryptic Soy Broth with 0.5% Glucose) to 1 × 10^6^ CFU/mL. Sub-MICs of ciprofloxacin were selected for the biofilm assay based on the MIC values of each isolate. The bacterial suspension was mixed with an equal volume of ciprofloxacin to obtain final concentrations of 2, 1, 0.5, 0.25, 0.125, 0.0625, and 0.03125 μg/mL. TSBG with 200 μL of the bacterial suspension without ciprofloxacin served as a negative control. To assess biofilm development, 24 h of incubation was selected as the timepoint, as this is commonly used in *S. aureus* biofilm studies (Periasamy et al., 2012). After incubation at 37°C for 24 h, excess medium was removed and adherent biofilms were washed twice with sterile PBS (Phosphate-Buffered Saline). Biofilms in the wells were fixed with 200 μL methanol for 15 min, air dried after removing the supernatant, and stained with 200 μL of 0.1% (w/v) crystal violet solution at room temperature for 5 min. After removing the stain, the wells were washed twice with sterile PBS. Then, 200 μL of 33% (v/v) acetic acid was added to each well to dissolve the stain. After shaking at room temperature for 30 min, OD_600_ was measured with Microplate Reader. The experiment was performed in triplicate with three replicates each time.

### 2.4 Confocal laser scanning microscope

CLSM assay was done as described before with minor modifications (Jin et al., 2020). *S. aureus* biofilms were prepared under similar culture conditions as described above in 20 mm glass bottom cell culture dishes. Biofilms were washed twice with sterile PBS, and then stained with 500 μL of fluorescent dye containing 0.02% SYTO 9 and 0.067% propidium iodide. Biofilm structure was observed using a CLSM system (Nikon, Tokyo, Japan). SYTO 9 dye was excited by a 488 nm argon laser and emission was collected at 520 nm. Propidium iodide was excited by a 559 nm argon laser and emission was collected at 619 nm. Live and dead bacteria fluoresced green and red, respectively.

### 2.5 Light microscopy observation of biofilm

The glass slide biofilm model was performed as described previously (Oates et al., 2014). Under the conditions described above, biofilms were cultured on sterile circular glass slides in 6-well plates. After incubation for 2 h, 24 h, and 48 h, slides with biofilms were gently washed with PBS and processed for Gram staining and imaged under a light microscope (Leica-DM2500, Leica Microsystems).

### 2.6 Initial attachment assay

The initial attachment assay was performed as described previously (Wang et al., 2023). Briefly, overnight cultures of *S. aureus* Newman were diluted 1:200 and inoculated into wells containing either 0.0625 μg/mL ciprofloxacin or no ciprofloxacin (control). After static incubation at 37°C for 2 h, unattached cells were discarded and wells were thoroughly washed 3 times with PBS. Attached cells were completely scraped and suspended in 1 mL sterile PBS, then counted by serial dilution. The experiment was performed in triplicate.

### 2.7 Growth curves

*S. aureus* strains were grown to exponential phase, then diluted 1:200 in TSB medium. Under different ciprofloxacin concentrations, bacteria were cultured with shaking at 220 rpm and 37°C. The OD_600_ of the bacterial cultures was measured every 1 h for 12 h. Experiments were performed in triplicate.

### 2.8 Quantification of polysaccharide intercellular adhesin

The quantification of PIA assay was conducted according to the previously described method (Rao et al., 2022). Briefly, bacteria were diluted 1:100 in 0.5% TSBG with or without ciprofloxacin and incubated in 6-well plates at 37°C for 24 h. Wells were then washed 3 times with PBS to remove planktonic bacteria. Biofilms of *S. aureus* with or without ciprofloxacin treatment were scraped and resuspended in 500 μL EDTA (0.5 M) (pH 8.0), boiled for 10 min, then centrifuged. Forty microliter of supernatant from each sample was digested with proteinase K (20 mg/mL) at 37°C for 3 h to reduce nonspecific background. PIA extracts were dotted onto methanol-activated PVDF membranes. After air drying, membranes were blocked with 5% skim milk at room temperature for 2 h, washed three times with PBST (0.1% Tween), then incubated with WGA-HRP at 37°C for 1 h. After three more PBST (0.1% Tween) washes, blots were detected and visualized using ECL Western blot substrate (Thermo Scientific, Rockford, IL, United States).

### 2.9 Autolysis assay

The autolysis assay was performed as described previously (Meehl et al., 2007). To determine the effect of ciprofloxacin on autolysis of *S. aureus*, log-phase cultures of both untreated (control) and ciprofloxacin-treated *S. aureus* (0.0625 μg/mL ciprofloxacin for Newman strain and 0.25 μg/mL ciprofloxacin for N315 strain) were centrifuged, washed twice with sterile distilled water, and resuspended in 50 mM Tris–HCl buffer (pH 7.5) containing 0.05% (v/v) Triton X-100 and the OD_600_ value was adjusted to 1.0. Suspensions were then incubated at 37°C with shaking at 220 rpm. Autolysis was monitored by measuring OD_600_ every 30 min for 3 h.

### 2.10 Proteolytic activity on agar plates

The proteolytic activity experiment was performed as described previously (Dotto et al., 2021). To assess proteolytic activity, 10 μL of supernatant from 24-h mature biofilms was spotted onto Tryptic Soy Agar (TSA) plates supplemented with 10% milk. After incubating at 37°C for 18 h, the diameters of proteolytic halos were measured using a caliper to indicate proteolytic activity. Repeat the experiment three times.

### 2.11 Real-time fluorescence quantitative PCR

To investigate the effect of ciprofloxacin on the transcriptional levels of biofilm-related genes and global regulators, RT-qPCR analysis was performed using *S. aureus* Newman. After treatment with or without 0.0625 μg/mL ciprofloxacin at 37°C in TSB for 24 h, bacteria were harvested by centrifugation and washed twice with cold saline. RNA was extracted using a total RNA purification kit (Sangon Biotech). RNA samples were reverse transcribed into cDNA using PrimeScript RT reagent kit (Takara Bio Inc., Dalian, China). qRT-PCR was carried out using TB GreenTM Premix (Takara) on a QuantStudioTM 5 Real-Time PCR System. Gene expression was normalized to *gyrB* levels and calculated by the ΔΔCt method (Livak and Schmittgen, 2001). Primers for biofilm-related genes and global regulators are listed in Supplementary Table S1. Each reaction was performed in triplicate.

### 2.12 Construction of gene deletion mutants and complementation mutants

The gene deletion mutants of Newman were constructed by homologous recombination using the plasmid pKOR1 as described previously (Bae and Schneewind, 2006), with minor modifications. Briefly, the upstream and downstream DNA fragments of *agrC* and *saeRS* (approx. 1,000 bp each) were amplified from Newman chromosomal DNA. The fused PCR products obtained by overlap extension PCR were cloned into pKOR1 vector by gateway BP clonase reaction using Gateway BP Clonase II (Thermo Fisher Scientific), generating recombinant plasmids pKOR1Δ*agrC* and pKOR1Δ*saeRS*. The obtained plasmids were then transferred into DH5α and DC10B, followed by electroporation into Newman. After chloramphenicol induction and incubation at 43°C, homologous recombination between the plasmid homology arms and the genome led to the deletion of *agrC* and *saeRS*. Finally, the plasmids were eliminated to obtain *agrC* and *saeRS* deletion mutants. For the *agrC* deletion mutant complementation, the full-length *agrC* gene and its native promoter region were PCR amplified and ligated into plasmid pLi50 using T4 ligase. The resulting complementation plasmid pLi50-*agrC* was electroporated into the *agrC* deletion mutant. Primers are listed in Supplementary Table S1.

### 2.13 Expression and purification of AgrC protein

The *S. aureus* Newman genome was extracted and used as a template. AgrC amplification primers were designed: upstream primer 5′- GTGGAATTATTAAATAGTT-3′, downstream primer 5′- GTTGTTAATAATTTCAACTT-3′. PCR was performed to obtain the *agrC* full-length gene fragment. The purified target gene was ligated with pBAD/Thio-TOPO vector (Napathorn et al., 2021). The ligation product was transformed into *E. coli* TOP10 competent cells by chemical transformation. Transformed bacteria were plated, positive clones were picked and identified. Single colonies containing the expression vector were picked from the stored plates, inoculated into LB medium containing ampicillin and cultured overnight. Plasmids were extracted and sequenced by Qingke Company. *E. coli* TOP10-pBAD/Thio-TOPO-*agrC* was cultured in LB medium (containing ampicillin), until OD_600_ reached 0.6. Arabinose was added to a final concentration of 0.002% and induction was performed at 180 r/min at 18°C. *E. coli* cells were harvested by centrifugation (12,000 g, 4°C, 5 min), resuspended in lysis buffer (20 mM Tris–HCl, pH 8.0, 1 mM dithiothreitol (DTT), and 0.5 M NaCl) containing PMSF (working concentration 1 mmol/L), and disrupted by low temperature high pressure cell disruptor (800 ~ 900 bar, repeated twice). After centrifugation (10,000 g, 4°C, 1 h), purification was performed using a nickel column chromatography system. Equilibration of the column and elution of the target protein were carried out using the following buffers: Equilibration buffer (20 mM Tris–HCl, pH 8.0, 1 mM DTT, 0.5 M NaCl) and Elution buffer (lysis buffer plus 300 mM imidazole). After elution, the protein was further dialyzed overnight against 2 L of dialysis buffer (20 mM Tris–HCl, pH 8.0, 1 mM DTT, and 50 mM NaCl).

### 2.14 Thermal shift assay

Thermal shift assays measuring changes in melting temperature (T_m_) can validate hypothesized interactions between test compounds and target proteins (Martinez Molina et al., 2013). To investigate the direct interaction between intracellular ciprofloxacin and AgrC protein, we performed CETSA experiments according to a published protocol (Martinez Molina et al., 2013). Different concentrations of ciprofloxacin (2, 4, 8, 16, 32, 64, 128 μg/mL) were incubated with AgrC protein (10 μM) at 37°C for 1 h. Then, supernatants were collected by centrifugation at 18,000 g, 4°C for 1 h. Equal amounts of supernatants were transferred to PCR tubes and heated at different temperatures (45.0, 48.0, 51.0, 54.0, 57.0 and 60.0°C) for 5 min using a PCR instrument, then immediately cooled on ice for 3 min. Supernatants were then obtained by 15,000 g centrifugation for 30 min and boiled for 5 min. Supernatants were analyzed by sodium dodecyl sulfate polyacrylamide gel electrophoresis (SDS-PAGE). The bands were visualized by staining with coomassie blue staining solution.

### 2.15 Molecular docking simulation analysis

The AgrC structure was derived from the 3D X-ray crystal structure in the Protein Data Bank (ID: 4BXI). The three-dimensional structure of ciprofloxacin was constructed using the ChemBio3D Ultra 12.0 software suite. Standard docking procedures for ciprofloxacin and AgrC protein were performed using AutoDock Tools v1.5.6 software (La Jolla, CA, United States) (Morris et al., 2009). Docking results were evaluated by ranking binding energies. Conformations with the lowest binding free energy were chosen as binding poses and visualized. Hydrogen bonds and hydrophobic interactions between ciprofloxacin and AgrC were visualized using Pymol 2.3.0 software (Lill and Danielson, 2011).

### 2.16 Statistical analysis

Data analyses were conducted using GraphPad Prism 8.0 software. Unpaired, two-tailed t-tests were employed to assess statistical significance, and a value of p of less than 0.05 was deemed to be statistically significant. Error bars on the graphs represented the standard deviation (mean ± SD).

## 3 Results

### 3.1 Subinhibitory ciprofloxacin enhances biofilm formation in both methicillin-sensitive and methicillin-resistant *Staphylococcus aureus*

**Figure 1:**
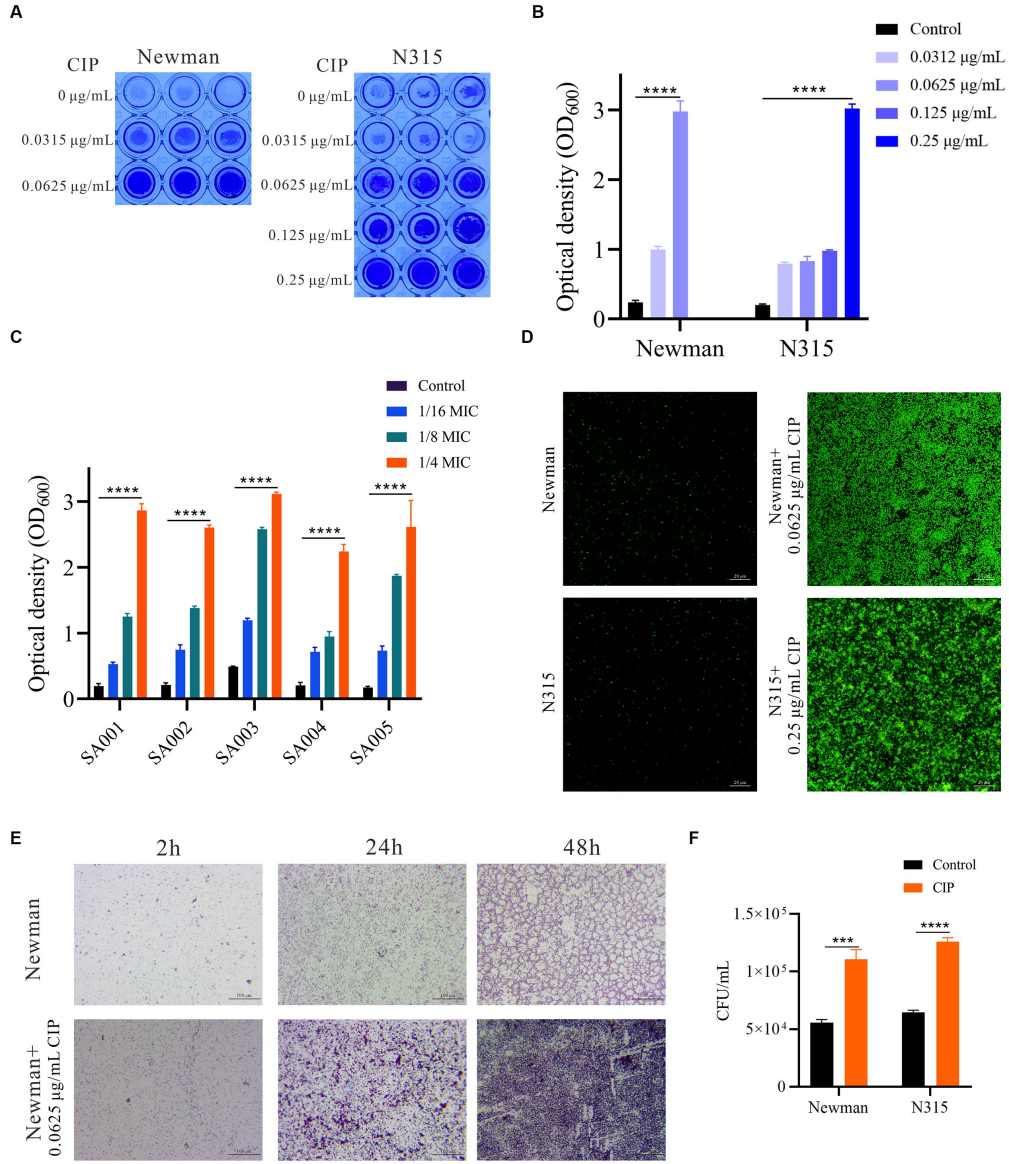
Subinhibitory ciprofloxacin induces dose-dependent biofilm formation in methicillin-sensitive and resistant *S. aureus*. **(A)** Representative images of crystal violet staining of biofilms formed by Newman and N315 strains in microtiter plate wells after 24 h of incubation. **(B)** Quantification of biofilm mass by measuring optical density at 600 nm (OD_600_) after acetic acid dissolution. Data represent mean ± SEM of triplicates. **(C)** Ciprofloxacin effect on clinical *S. aureus* isolate biofilm formation after 24 h of incubation. **(D)** CLSM images of biofilms treated with 0.0625 μg/mL Ciprofloxacin for Newman strain and 0.25 μg/mL for N315 strain after 24 h of incubation. Images were acquired using a 63x glycerol immersion objective. The scale bar is 20 μm. **(E)** Light micrographs of Newman strain slide biofilms treated with 0.0625 μg/mL ciprofloxacin compared to untreated biofilms at 2, 24, and 48 h. Images were acquired under light microscope at 100x magnification. The scale bar is 100 μm. **(F)** Quantification of slide-adherent for the Newman and N315 strains cells by CFU counting after 2 h incubation with or without ciprofloxacin (0.0625 μg/mL ciprofloxacin-treated Newman and 0.25 μg/mL ciprofloxacin-treated N315). *p < 0.05; **p < 0.01; ***p < 0.001; ****p < 0.0001.

The MIC of ciprofloxacin was 0.25 μg/mL for *S. aureus* Newman (MSSA) and 1 μg/mL for N315 (MRSA). The crystal violet staining method was utilized to evaluate the impact of varying concentrations of ciprofloxacin on biofilm formation in two representative laboratory strains, Newman (MSSA) and N315 (MRSA). As shown in Figure 1A, biofilm biomass increased in a dose-dependent manner with ciprofloxacin exposure in both Newman and N315 strains. At a concentration of 0.0625 μg/mL, ciprofloxacin induced a remarkable 12.46-fold increase in Newman biofilm biomass, while at 0.25 μg/mL, N315 biofilm biomass was enhanced by 15.19-fold (Figure 1B). Given clinical isolates with diverse genetic backgrounds may exhibit varying biofilm phenotypes, we investigated the effects of ciprofloxacin on biofilm formation in five clinical *S. aureus* isolates, including 2 MSSA and 3 MRSA, representing different multilocus sequence types as detailed in Table 1. Overall, a consistent trend was observed where subinhibitory concentrations of ciprofloxacin stimulated *S. aureus* biofilm formation across isolates (Figure 1C).

To further visualize the biofilm-promoting effect of ciprofloxacin, CLSM analysis was conducted on *S. aureus* Newman and N315 strains. The CLSM results consistently revealed significantly denser and more compact biofilms populated with an increased number of live cells following ciprofloxacin treatment (Figure 1D). Collectively, these findings provide strong evidence that subinhibitory concentrations of ciprofloxacin potently enhances *S. aureus* biofilm formation.

Bacterial biofilm development involves three phases: attachment, maturation, and dispersal. To determine which phase was influenced by ciprofloxacin, we visualized Newman slide biofilms at three different time points during 0.0625 μg/mL ciprofloxacin treatment (Figure 1E). Strikingly, light micrographs with Gram staining demonstrated the induction of biofilm formation by ciprofloxacin at all examined biofilm stages. During the attachment phase (2 h), more bacterial cells adhered and formed aggregates with greater density on ciprofloxacin-exposed slides. Colony-forming unit (CFU) counting revealed a 1.94-fold to 1.99-fold increase in slide-adherent cells for both the Newman and N315 strains in response to ciprofloxacin (Figure 1F). In both the maturation (24 h) and dispersal (48 h) phases, slide biofilms also exhibited pronounced increases in microcolonies and biofilm matrix compared to respective untreated controls (Figure 1E).

### 3.2 Ciprofloxacin does not impact growth but enhances PIA synthesis of *Staphylococcus aureus*

**Figure 2:**
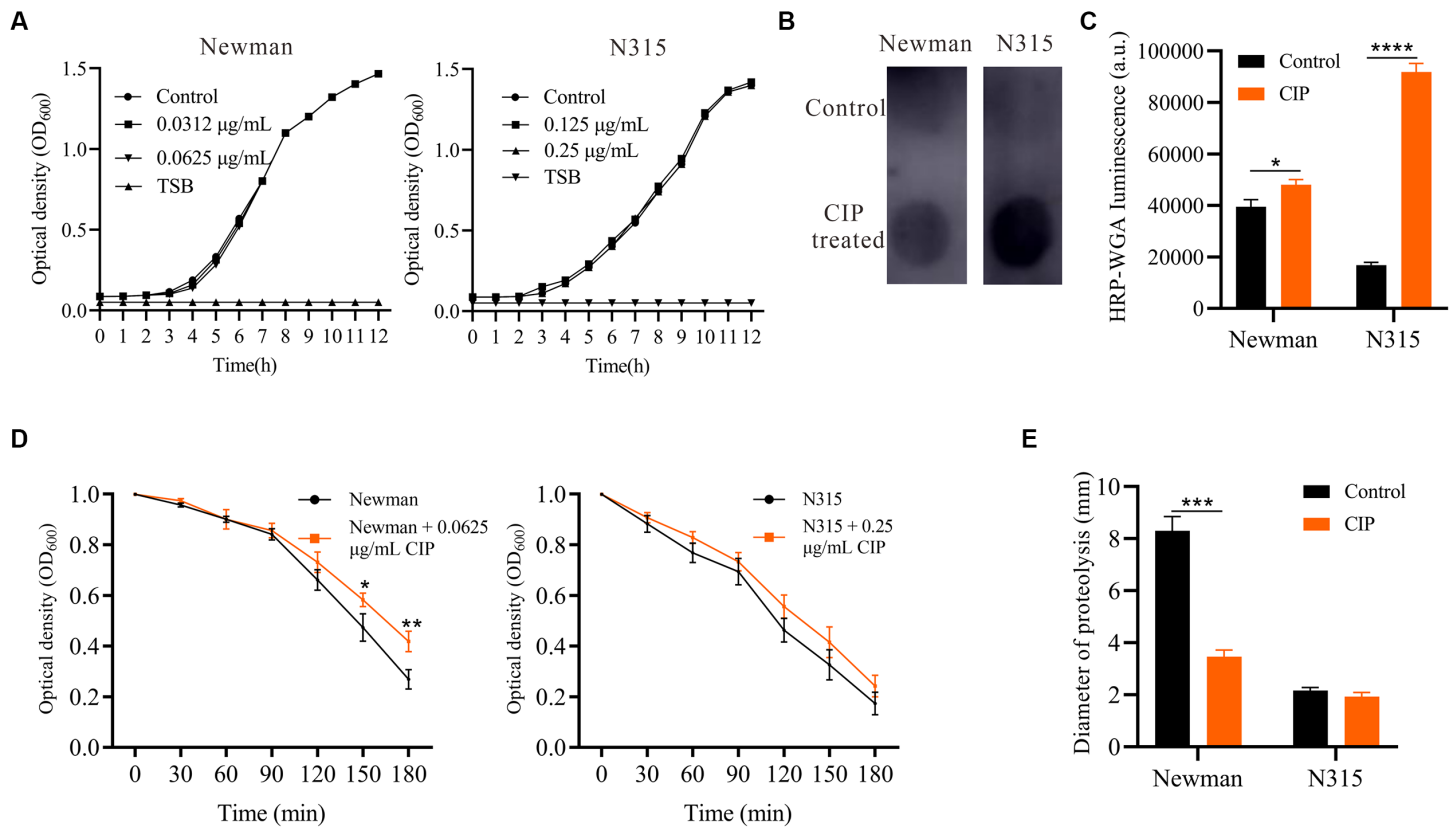
Impact of Ciprofloxacin on *S. aureus* growth, PIA production, autolysis and proteolytic activity. **(A)** Growth curves of ciprofloxacin-treated versus untreated Newman and N315. **(B)** PIA production in ciprofloxacin-treated versus untreated *S. aureus* measured by dot blot assay (0.0625 μg/mL ciprofloxacin-treated Newman and 0.25 μg/mL ciprofloxacin-treated N315). **(C)** Bar graphs show the quantitative values of the signal intensity, measured in arbitrary units (a.u.), using ImageJ. **(D)** Triton X-100 induced autolysis of ciprofloxacin-treated versus untreated Newman and N315 monitored by decrease in OD_600_ over time (0.0625 μg/mL ciprofloxacin-treated Newman and 0.25 μg/mL ciprofloxacin-treated N315). **(E)** Proteolytic activity of supernatants of ciprofloxacin-treated versus untreated *S. aureus* biofilms (0.0625 μg/mL ciprofloxacin-treated Newman and 0.25 μg/mL ciprofloxacin-treated N315). *p < 0.05; **p < 0.01; ***p < 0.001; ****p < 0.0001.

To preclude the possibility that ciprofloxacin enhances biofilm formation by increasing bacterial growth rate, we evaluated the effect of subinhibitory concentrations of ciprofloxacin (0.0625 μg/mL for Newman and 0.25 μg/mL for N315) on the growth kinetics of *S. aureus* using growth curves (Figure 2A). Remarkably, these concentrations of ciprofloxacin did not significantly affect the growth of either Newman or N315 strains. This suggests that ciprofloxacin does not promote biofilm formation in *S. aureus* through the stimulation of bacterial growth. To determine whether PIA is involved in ciprofloxacin-induced biofilm formation, we extracted and quantified PIA using a dot blot assay (Figure 2B). The results revealed a mild increase in PIA levels in ciprofloxacin-treated Newman compared to the untreated control (*p* = 0.01). Notably, there was a significantly higher level of PIA in the ciprofloxacin-treated N315 strain compared to the untreated control (*p* < 0.001) (Figure 2C). These findings indicate that ciprofloxacin can indeed enhance PIA synthesis in *S. aureus*, leading to the development of a biofilm matrix with a higher PIA content.

### 3.3 Ciprofloxacin slows autolysis of *Staphylococcus aureus* and decreases proteolysis activity of the biofilms

To investigate the impact of ciprofloxacin on *S. aureus* autolysis, we performed Triton X-100 autolysis assays. Ciprofloxacin-treated *S. aureus* Newman displayed significantly lower autolysis kinetics compared to untreated cells at 2.5 h and 3 h (Figure 2D). Autolysis was mildly reduced in ciprofloxacin-treated N315, but the difference was not statistically significant (*p* > 0.05). Thus, ciprofloxacin may stabilize biofilms by attenuating *S. aureus* lysis and lowering autolytic channels within the matrix, which could explain the enhanced adhesion and biofilm formation. Since proteases also degrade cell surface proteins and matrix components, impairing biofilm stability (Paharik and Horswill, 2016), we examined protease activity in ciprofloxacin-treated *S. aureus* biofilm supernatants using milk agar plates. The ciprofloxacin-treated biofilms of the Newman strain showed reduced proteolysis halos, indicating a decrease in protease activity (Figure 2E). However, there were no significant differences observed in the proteolysis halos of the ciprofloxacin-treated N315 strain biofilms.

### 3.4 Ciprofloxacin exposure impacts transcription of biofilm-related genes and transcriptional regulatory genes

**Figure 3:**
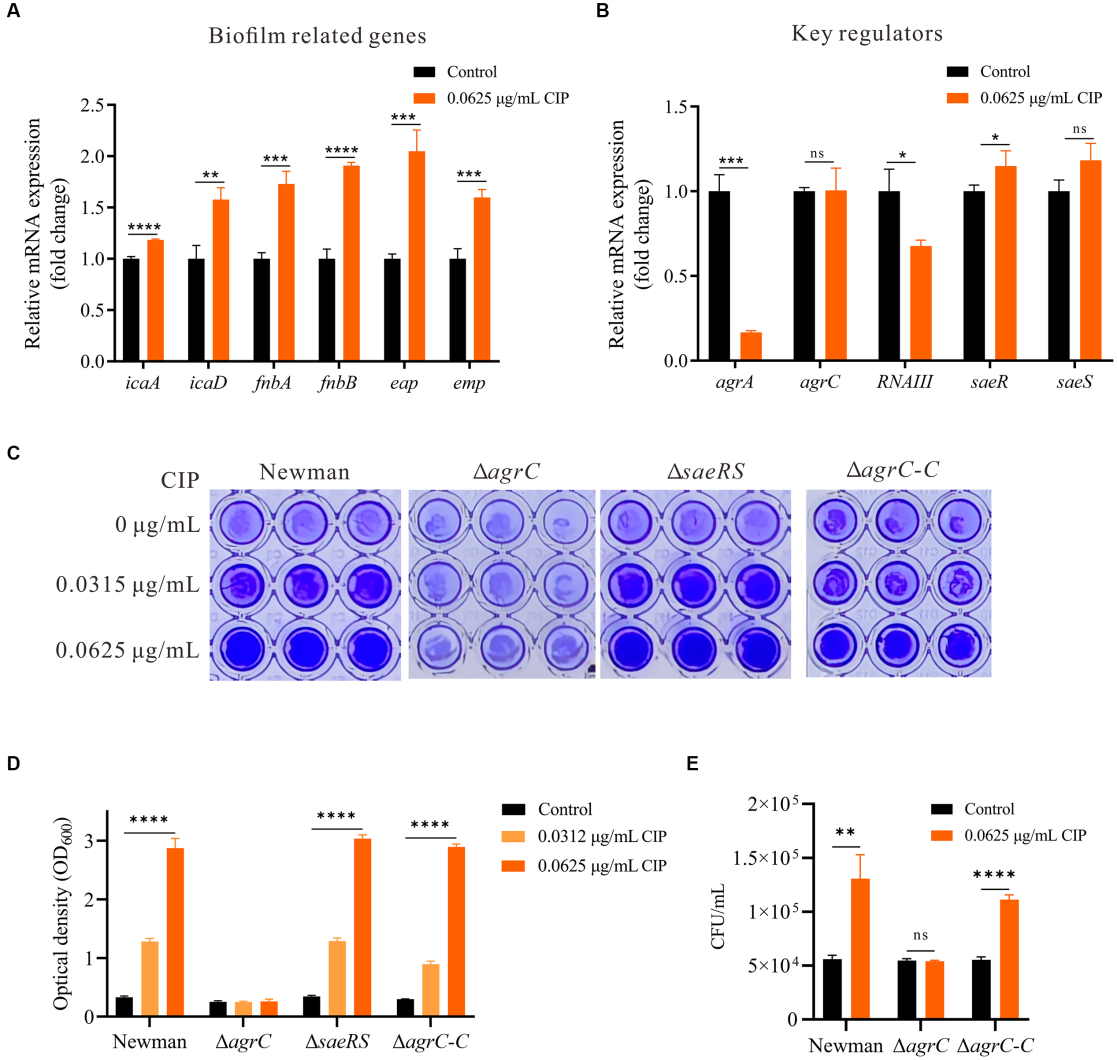
Mechanisms underlying the enhancement of biofilm formation by ciprofloxacin. **(A)** RT-qPCR revealed ciprofloxacin impacts the expression level of genes related to biofilm formation in Newman. **(B)** RT-qPCR revealed ciprofloxacin impacts the expression level of key regulators in Newman. **(C)** Effect of ciprofloxacin on biofilm formation in Newman, *agrC* Mutant, *saeRS* Mutant, and *agrC* Complemented Strain. **(D)** Quantification of biofilm mass by OD_600_ after acetic acid dissolution. **(E)** Enumeration of initial adherent cells (2 h) for ciprofloxacin-treated Newman, Δ*agrC*, and Δ*agrC*-C strains. *p < 0.05; **p < 0.01; ***p < 0.001; ****p < 0.0001.

To elucidate mechanisms of ciprofloxacin-enhanced biofilms, we used RT-qPCR to analyze the expression of genes related to biofilm formation. Ciprofloxacin significantly increased *icaA*, *icaD*, *fnbA*, *fnbB*, *eap*, and *emp* expression (*p* < 0.05) (Figure 3A). *S. aureus* biofilm formation is controlled by several global regulators such as Agr and Sae (Paharik and Horswill, 2016). Therefore, we hypothesized ciprofloxacin may enhance *S. aureus* biofilm formation by altering *agr* or *sae*. We investigated the expression of genes related to the Agr system (*agrA*, *agrC* and *RNAIII*) and the Sae system (*saeR* and *saeS*) during biofilm formation at 24 h, both in the presence and absence of ciprofloxacin. Our results demonstrate that with ciprofloxacin treatment, the expression level of *agrA* and *RNAIII* were significantly lower than those in the control group (*p* < 0.05) and *agrC* was not significantly different (Figure 3B). Additionally, compared to the control group, the expression levels of *saeR* in the presence of ciprofloxacin exhibited a slight upregulation (*p* = 0.045).

### 3.5 Ciprofloxacin promotes *Staphylococcus aureus* biofilm formation in an *agrC*-dependent manner

Based on our findings, we reasoned ciprofloxacin may not directly enhance *S. aureus* biofilm formation by impacting PIA production, autolysis, and/or proteolysis activity, but rather through influencing global regulators in *S. aureus*. To investigate which global regulator mediated the mechanism of ciprofloxacin-enhanced biofilm formation, we generated *agrC* and *saeRS* deletion mutants for comparative measurements. The results demonstrated that ciprofloxacin-induced biofilm formation enhancement was significantly inhibited in the *agrC* deletion mutant, while no obvious change occurred in the *saeRS* mutant. Additionally, we constructed an *agrC* complementation mutant and studied its response to ciprofloxacin. When the *agrC* complement was cultured with ciprofloxacin, an expected increase in biofilm accumulation was observed at a level comparable to the WT strain (Figures 3C,D). Moreover, deletion and complement of *agrC* also clearly impaired and restored bacteria initial attachment in response to ciprofloxacin, respectively (Figure 3E). Therefore, we conclude ciprofloxacin-induced biofilm formation is mediated in an *agrC*-dependent manner in *S. aureus*.

### 3.6 Thermal stability assay of AgrC protein treated with ciprofloxacin

**Figure 4:**
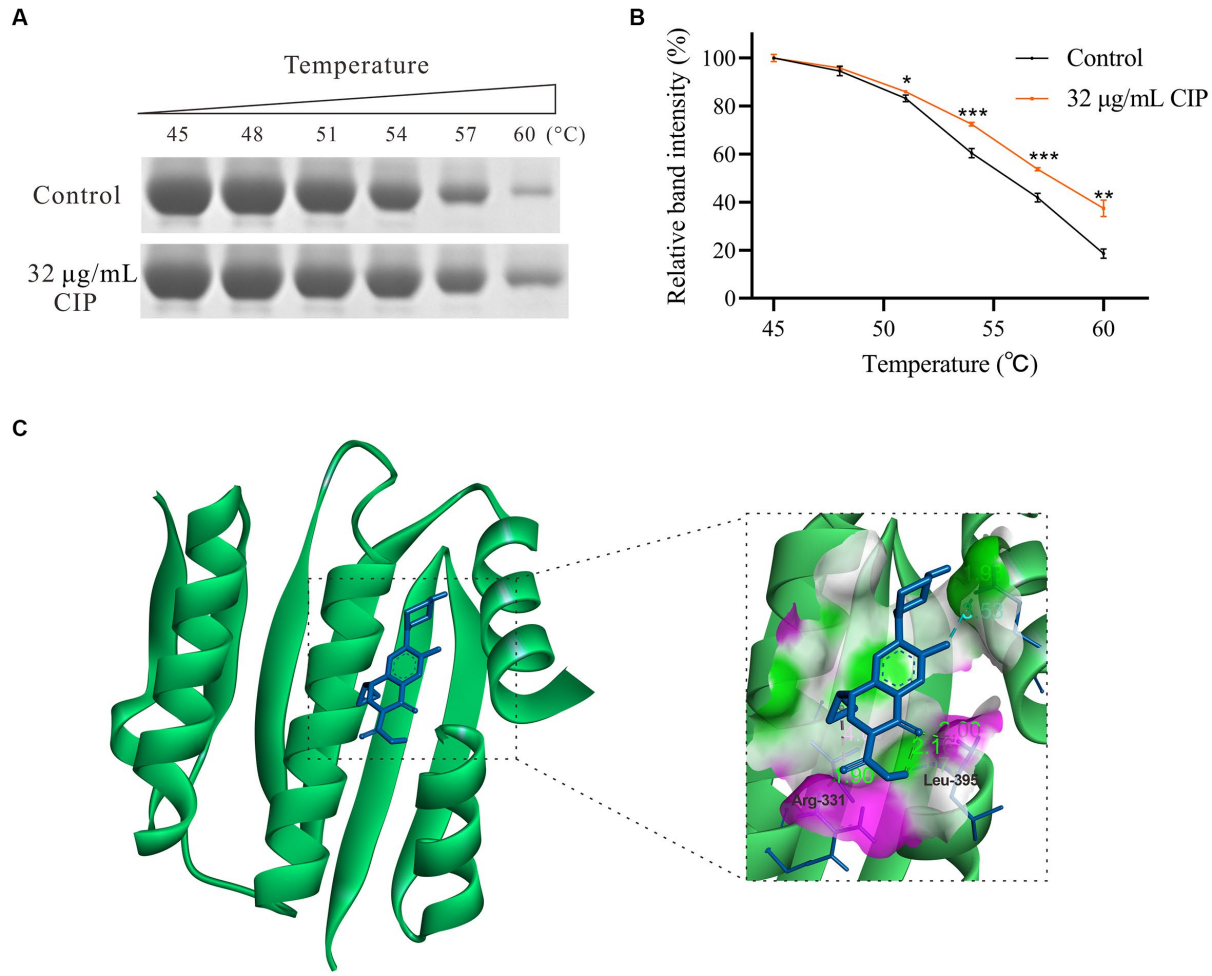
Interaction between Ciprofloxacin and the AgrC Protein. **(A)** Thermal shift assay showing ciprofloxacin (32 μg/mL) stabilization of AgrC protein across a temperature gradient and subsequent SDS-PAGE analysis. **(B)** Grayscale quantification of SDS-PAGE bands. **(C)** Molecular modeling of the AgrC-ciprofloxacin binding. *p < 0.05; **p < 0.01; ***p < 0.001; ****p < 0.0001.

RT-qPCR results indicate that subinhibitory concentrations of ciprofloxacin did not significantly impact the expression level of the *agrC* (*p* = 0.109). However, it notably reduced the expression levels of its target genes, *agrA*, as well as the downstream gene, *RNAIII*. Therefore, we propose that ciprofloxacin may have the capability to directly bind to the AgrC protein, thereby inhibiting the transcription of downstream genes regulated by the Agr system. Thus, His-tagged AgrC protein was successfully expressed and purified. Thermal stability assays were then performed, incubating AgrC protein with 32 μg/mL ciprofloxacin across a temperature gradient. Our data showed ciprofloxacin significantly increased AgrC thermal stability, evidenced by higher T_m_ shifts versus untreated protein (Figures 4A,B). This demonstrates ciprofloxacin binds and stabilizes AgrC protein.

### 3.7 Molecular docking simulation analysis

To further characterize the ciprofloxacin-AgrC interaction, we predicted their binding site using molecular docking simulation analysis. The modeling revealed two hydrogen bonds between ciprofloxacin and key AgrC active site residues Arg331 (1.90 Å) and Leu395 (2.00 Å) (Figure 4C). The binding energy was calculated to be −6.54 kcal/mol, indicating favorable binding. Collectively, thermal shift and docking data strongly support that ciprofloxacin directly binds AgrC to modulate downstream signaling and thus enhance *S. aureus* biofilm formation.

## 4 Discussion

Numerous studies have demonstrated that subinhibitory concentrations of various antibiotics can enhance biofilm formation by *S. aureus* (Hoffman et al., 2005). Ciprofloxacin is a broad-spectrum antibiotic with activity against many Gram-positive and Gram-negative bacteria that have developed resistance to other antibiotics (Sharma et al., 2017). This characteristic endows ciprofloxacin with a notable advantage in managing polymicrobial infections. While previous studies have demonstrated that subinhibitory concentrations of ciprofloxacin decrease biofilm formation in *Pseudomonas aeruginosa* and *S. epidermidis*, its impact on biofilm formation in *S. aureus* and the underlying mechanisms remain unclear (Gupta et al., 2016; Szczuka et al., 2017). Here, we demonstrate that ciprofloxacin significantly enhances *S. aureus* biofilm formation independent of methicillin resistance in an *agrC*-dependent manner.

The extracellular matrix composed of polysaccharides like PIA, eDNA, and proteins mediates multicellular interactions during *S. aureus* biofilm formation (Paharik and Horswill, 2016). PIA, synthesized by the *icaADBC* operon, is an integral matrix component (Nguyen et al., 2020). Additionally, *S. aureus* catalyzes self-lysis and component release through murein hydrolase-mediated cell wall degradation, liberating substantial eDNA that reinforces the biofilm matrix (Secchi et al., 2022). Previous studies demonstrate some antibiotics like ceftaroline and oxacillin can increase *icaA* expression (Jo and Ahn, 2016; Lázaro-Díez et al., 2016), indicating PIA may be involved in the antibiotic modulation of bacterial biofilms. It is widely accepted that MRSA strains can form biofilms via PIA-independent mechanisms (McCarthy et al., 2015). In this study, ciprofloxacin induced a significant increase in PIA levels in MRSA strain N315, but only a slight increase in PIA levels in MSSA strain Newman. This indicates that ciprofloxacin can stimulate PIA synthesis to modulate the *S. aureus* biofilm matrix, regardless of the bacterium’s methicillin susceptibility status. Increased PIA and extracellular matrix reinforcement within ciprofloxacin-induced biofilms could enhance biofilm stability and facilitate bacterial persistence over time (Nguyen et al., 2020). Denser biofilm structures may provide physically protected niches that better shield embedded cells from antibiotic exposure and host immune defenses (Boles and Horswill, 2008). This could promote the establishment of chronic, refractory infections where residual biofilm-associated bacteria continuously seed recurrent acute infection even during antibiotic treatment.

The Agr quorum sensing system plays an important role in *S. aureus* biofilm formation by regulating the delicate balance between autolysis and biofilm stability (Yarwood et al., 2004; Boles and Horswill, 2008). Excessive autolysis causes premature dispersal (Bayles, 2007; Rice et al., 2007; Qamar and Golemi-Kotra, 2012). Previous studies have shown that *agr* mutants or inhibition reduces autolysis in *S. aureus* (Fujimoto and Bayles, 1998; Dotto et al., 2021). When active, *agr* stimulates protease expression and downregulates adhesins, attenuating aggregation and impeding biofilm formation (Yarwood et al., 2004; Boles and Horswill, 2008). This implies that ciprofloxacin may enhance biofilm formation by downregulating the Agr system. Thus, we investigated the effect of ciprofloxacin on *S. aureus* autolysis and proteolysis activity. We found that ciprofloxacin inhibited Newman autolysis and lowered proteolytic activity in biofilms. Although the N315 strain, treated with ciprofloxacin, exhibited only a mild reduction in autolysis rates, the difference did not reach statistical significance. However, this may be attributed to the fact that the N315 strain, as a MRSA, possesses a thicker cell wall in comparison to the Newman strain (MSSA). The variations in autolysis rates between these strains can be ascribed to differences in cell wall composition and the types and quantities of autolysins present. Additionally, N315 strain demonstrate lower proteolytic activity compared to Newman strain. This diversity may stem from various factors, including genetic variations associated with methicillin resistance, disparities in the regulation of virulence factors, evolutionary adaptations, and strain-specific characteristics (Singh and Phukan, 2019). These results highlight the intricate nature of ciprofloxacin’s impact on biofilm formation and emphasize the significance of considering strain-specific characteristics. Transcriptionally, the major *agr* transcripts (*RNAIII* and *agrA*) were significantly downregulated in strain Newman after ciprofloxacin treatment. These results support the hypothesis that ciprofloxacin negatively regulates *agr* expression.

The Agr operon encodes the propeptide AgrD, which is processed into the autoinducing peptide AIP. When AIP binds to AgrC above a threshold density, it modulates AgrC signaling and downstream gene expression (Yarwood et al., 2004; Kavanaugh and Horswill, 2016). This triggers phosphorylation cascades in the Agr quorum sensing system, ultimately activating *RNAIII* to regulate *S. aureus* biofilm formation and dispersal (Williams et al., 2023). As the key receptor, AgrC likely plays a critical regulatory role in biofilm development. Indeed, deletion and complementation of *agrC* directly demonstrated its critical regulatory role in ciprofloxacin’s inductive effect on biofilm enhancement (Figures 3C,D). Although ciprofloxacin upregulated expression of *saeR* and Sae system target genes (*eap* and *emp*), the *saeRS* mutant maintained ciprofloxacin-stimulated biofilms (Figure 3C). Collectively, these results indicate demonstrate *agrC* is indispensable for ciprofloxacin-induced biofilm enhancement. Since ligand-protein binding is often discovered in more complex settings, thermal shift assays can further validate drug-target interactions (Martinez Molina et al., 2013). Our results demonstrate ciprofloxacin can binds AgrC protein, increasing its thermal stability. Molecular docking reveals ciprofloxacin may bind key AgrC active site residues Arg331 and Leu395. Inhibition of AgrC function by ciprofloxacin binding may lower the quorum sensing threshold for downstream signaling activation or disrupt proper phosphorylation cascades mediated by AgrC. Future studies employing site-directed mutagenesis experiments to validate the amino acid binding site between ciprofloxacin and AgrC are necessary to elucidate the precise molecular mechanism.

Overall, these results lead us to hypothesize that ciprofloxacin may competitively bind to AgrC, displacing AIP. Peterson et al. reported that the lipoprotein apoB in human serum antagonizes AgrC signaling by blocking AIP binding (Peterson et al., 2008). Ciprofloxacin may function through an analogous mechanism, also interfering with AIP-AgrC interaction. Furthermore, ciprofloxacin may synergize with plasma components *in vivo* to promote conditions that downregulate *agr* expression. The few cells with attenuated quorum sensing escaping the biofilm may have an advantage in adhering and forming new stable biofilms elsewhere. Moreover, ciprofloxacin-induced *agr* dysfunction favors chronic infection. Escaped cells capable of forming new biofilms under ciprofloxacin selective pressure could contribute to biofilm-associated bacterial persistence and chronic infection recurrence even during antimicrobial therapy. Given the common prescribing of fluoroquinolones for polymicrobial infections, their potential to unintentionally facilitate *S. aureus* biofilm lifestyle through this mechanism may compromise infection clearance. Therefore, caution is warranted regarding subinhibitory concentrations of ciprofloxacin exposures to avoid exacerbating recalcitrant, multidrug-resistant infections.

In conclusion, our findings demonstrate that ciprofloxacin promotes *S. aureus* biofilm formation by binding and inhibiting the AgrC receptor to modulate downstream signaling. This work provides initial mechanistic insights into how subinhibitory concentrations of ciprofloxacin enhance *S. aureus* antibiotic resistance.

## Data availability statement

The original contributions presented in this study are included in the article/Supplementary material, further inquiries can be directed to the corresponding author.

## Author contributions

Z-xL: Writing – original draft, Formal analysis, Investigation, Project administration, Validation. YL: Data curation, Writing – original draft. M-fL: Data curation, Methodology, Writing – original draft. RZ: Funding acquisition, Writing – review & editing, Conceptualization.
