# Supplementary material for: Ciprofloxacin enhances the biofilm formation of Staphylococcus aureus via an agrC-dependent mechanism
Source: Front Microbiol. 2023 Dec 21;14:1328947. doi: 10.3389/fmicb.2023.1328947 (PMC10764545; doi:10.3389/fmicb.2023.1328947)
Supplement: Supplementary file 1 [file Data_Sheet_1.DOCX]

Supplementary Material

**Ciprofloxacin Enhances the biofilm formation of *Staphylococcus aureus* via an *agrC*-dependent mechanism**

First author: Zhao-xia Luo and Yuting Li

Correspondence: Rui Zhao, [zhaoruisc@163.com](mailto:zhaoruisc@163.com)

Journal: *Frontiers in microbiology*

**Supplementary Material**

Supplementary Table S1. Primers used in this study.

| Primer name | Sequence (5'-3') | Target | Source or reference |
| --- | --- | --- | --- |
| *gyrB*-RT-F | ACATTACAGCAGCGTATTAG | For qRT-PCR | Rao et al. 2022 |
| *gyrB*-RT-R | CTCATAGTGATAGGAGTCTTCT |  |  |
| *icaA*-RT-F | GTTGGTATCCGACAGTATA |  | Rao et al. 2022 |
| *icaA*-RT-R | CACCTTTCTTACGTTTTAATG |  |  |
| *icaD*-RT-F | ATGGTCAAGCCCAGACAGAG |  | Kouidhi et al. 2010 |
| *icaD*-RT-R | AGTATTTTCAATGTTTAAAGCA |  |  |
| *fnbA*-RT-F | TTCCTTAACTACCTCTTCT |  | This study |
| *fnbA*-RT-R | CAATCATATAACGCAACAG |  |  |
| *fnbB*-RT-F | ACGACTGGTTGTTAGGTT |  | This study |
| *fnbB*-RT-R | GTGAGACTACGGTTAGCA |  |  |
| *eap*-RT-F | TCTATATCACTAATACCTCTGTCT |  | This study |
| *eap*-RT-R | CATCAAGTTCGTTACACCAT |  |  |
| *emp*-RT-F | ACCACCACTTCATTACTAC |  | This study |
| *emp*-RT-R | AAGATTGAGATGGATGTGTATA |  |  |
| *agrA*-RT-F | GCAGTAATTCAGTGTATGTTCA |  | Rao et al. 2022 |
| *agrA*-RT-R | TATGGCGATTGACGACAA |  |  |
| *agrC*-RT-F | CTGGCCTACGTGATTA |  | This study |
| *agrC*-RT-R | GCTACTTACTTCATCGGG |  |  |
| *RNAIII*-RT-F | TTCACTGTGTCGATAATCCA |  | This study |
| *RNAIII*-RT-R | TGATTTCAATGGCACAAGAT |  |  |
| *saeR*-RT-F | GTCGTAACCATTAACTTCTG |  | Rao et al. 2022 |
| *saeR*-RT-R | ATCGTGGATGATGAACAA |  |  |
| *saeS*-RT-F | CGTTCTTGTAGTTCTGGTAT |  | This study |
| *saeS*-RT-R | GTTGGTAGTCGCATTGATA |  |  |
| *agrC*-up-F | GGGGACAAGTTTGTACAAAAAAGCAGGCTCTAAGTCACCGATTGTTG | For isogenic deletion of *agrC* in Newman | This study |
| *agrC*-up-R | ACCTTCACCTTTAGTAGATTAATAATTCCACCTACTATCAC |  |  |
| *agrC*-Down-F | GTGATAGTAGGTGGAATTATTAATCTACTAAAGGTGAAGGT |  | This study |
| *agrC*-Down-R | GGGGACCACTTTGTACAAGAAAGCTGGGTAATTGAATACGCCGTTAA |  |  |
| *saeRS*-up-F | GGGGACAAGTTTGTACAAAAAAGCAGGCTCTTGCCACAATAGATAGG | For isogenic deletion of *saeRS* in Newman | This study |
| *saeRS*-up-R | ATGATGCCAGAAGTTAATTAGGAACTACGATGACTG |  |  |
| *saeRS*-Down-F | CAGTCATCGTAGTTCCTAATTAACTTCTGGCATCAT |  | This study |
| *saeRS*-Down-R | GGGGACCACTTTGTACAAGAAAGCTGGGTTACATTACACTCATTACTCA |  |  |
| *agrC*-C-F | CGCGGATCCCATTATCACACTTATCATCA | For *agrC* complement in NewmanΔ*agrC* | This study |
| *agrC*-C-R | CCCAAGCTTTCATACATTCACATCCTT |  |  |

# References:

Kouidhi, B., Zmantar, T., Hentati, H., and Bakhrouf, A. (2010). Cell surface hydrophobicity, biofilm formation, adhesives properties and molecular detection of adhesins genes in *Staphylococcus aureus* associated to dental caries. *Microb. Pathog*. 49, 14–22. doi: 10.1016/j.micpath.2010.03.007.

Rao, L., Yu, J., Wang, B., Zhao, H., Wang, X., Guo, Y., et al. (2022). Small-Molecule Compound SYG-180-2-2 to Effectively Prevent the Biofilm Formation of Methicillin-Resistant *Staphylococcus aureus*. *Front. Microbiol*. 12, 770657. doi: 10.3389/fmicb.2021.770657.
